# Quantitative control of mitochondria transfer between live single cells using a microfluidic device

**DOI:** 10.1242/bio.024869

**Published:** 2017-11-01

**Authors:** Ken-Ichi Wada, Kazuo Hosokawa, Yoshihiro Ito, Mizuo Maeda

**Affiliations:** 1 Bioengineering Laboratory, RIKEN, 2-1 Hirosawa, Wako, Saitama 351-0198, Japan; 2 Nano Medical Engineering Laboratory, RIKEN, 2-1 Hirosawa, Wako, Saitama 351-0198, Japan

**Keywords:** Cell fusion, Mitochondrial cloning, Homoplasmic mutation of mtDNA

## Abstract

Quantitative control of mitochondria transfer between live cells is a promising approach for genetic manipulation of mitochondrial DNA (mtDNA) because single mitochondrion transfer to a mtDNA-less (ρ^0^) cell potentially leads to homoplasmy of mtDNA. In this paper, we describe a method for quantitative control of mitochondria transfer between live single cells. For this purpose, we fabricated novel microfluidic devices having cell paring structures with a 4.1, 5.6 or 10.0 μm-length microtunnel. When cells were fused through a microtunnel using the Sendai virus envelope-based method, a strictured cytoplasmic connection was achieved with a length corresponding to that of the microtunnel. Elongation of the cytoplasmic connection led to a decrease in mitochondria transfer to the fusion partner. Moreover, some cell pairs that fused through a 10.0 μm-length microtunnel showed single mitochondrion transfer. Fused cells were spontaneously disconnected from each other when they were recovered in a normal culture medium. These results suggest that our cell fusion method can perform quantitative control of mitochondria transfer that includes a single mitochondrion transfer.

## Abstract (precis)

**Summary:** We developed a novel mitochondria transfer platform using a microfluidic device, and succeeded in single mitochondrion transfer between live single cells.

## INTRODUCTION

Mitochondria have their own genome, or mitochondrial DNA (mtDNA), encoding subunits of the oxidative phosphorylation enzyme complex, and also tRNAs and rRNAs for their translation. A cell contains several thousands copies of mtDNA, and dysfunctions of the mutated mtDNA are compensated by other mtDNAs existing in the same cell (Ono et al., 2001; Nakada et al., 2001). Therefore, for functional analysis of mtDNA, introducing the same mutation(s) to all copies of mtDNA (i.e. achievement of homoplasmy of mutated mtDNA) is required; however, convenient methods for the genetic manipulation of mtDNA are not available.

Despite the absence of convenient methods, previous studies have succeeded in achieving homoplasmic mutations of mtDNA in limited situations. It has been reported that removal of non-mutated mtDNA from heteroplasmic cells by mitochondria-targeting nucleases can achieve homoplasmy of mutated mtDNA (Xu et al., 2008); however, this method has a limitation concerning mutation design and risks interfering with the nuclear genome. The chemical elimination of mtDNA, such as exposure to ethidium bromide, also has the potential to achieve homoplasmy. This approach involves homoplasmy arising from heteroplasmic cells by reducing mtDNA copy number (ideally by a single copy in a cell) and subsequent mtDNA recovery (Acín-Pérez et al., 2004; Moreno-Loshuertos et al., 2006). Theoretically, this method potentially makes any mtDNA mutations contained in the cell homoplasmic; however, its throughput is low because of the difficulty concerning proper elimination of mtDNA.

Mitochondria segregation by cell fusion with a mtDNA-less (ρ^0^) cell is an another promising approach for the achievement of mutated mtDNA homoplasmy. Repeated cytoplast (enucleated cell) fusion with ρ^0^ cells could make a highly accumulated mtDNA mutation homoplasmic (Ono et al., 2001). Moreover, synaptosome (small cellular fragment from neuron) fusion with a ρ^0^ cell potentially achieves homoplasmy of a minor population of mutated mtDNA (Trounce et al., 2000; McKenzie et al., 2014), perhaps due to the transfer of a small number of mitochondria to the ρ^0^ cell. This strongly suggests that single mitochondrion transfer to a ρ^0^ cell, or mitochondrial cloning, is a reliable approach to achieve mutated mtDNA homoplasmy.

**Fig. 1.**
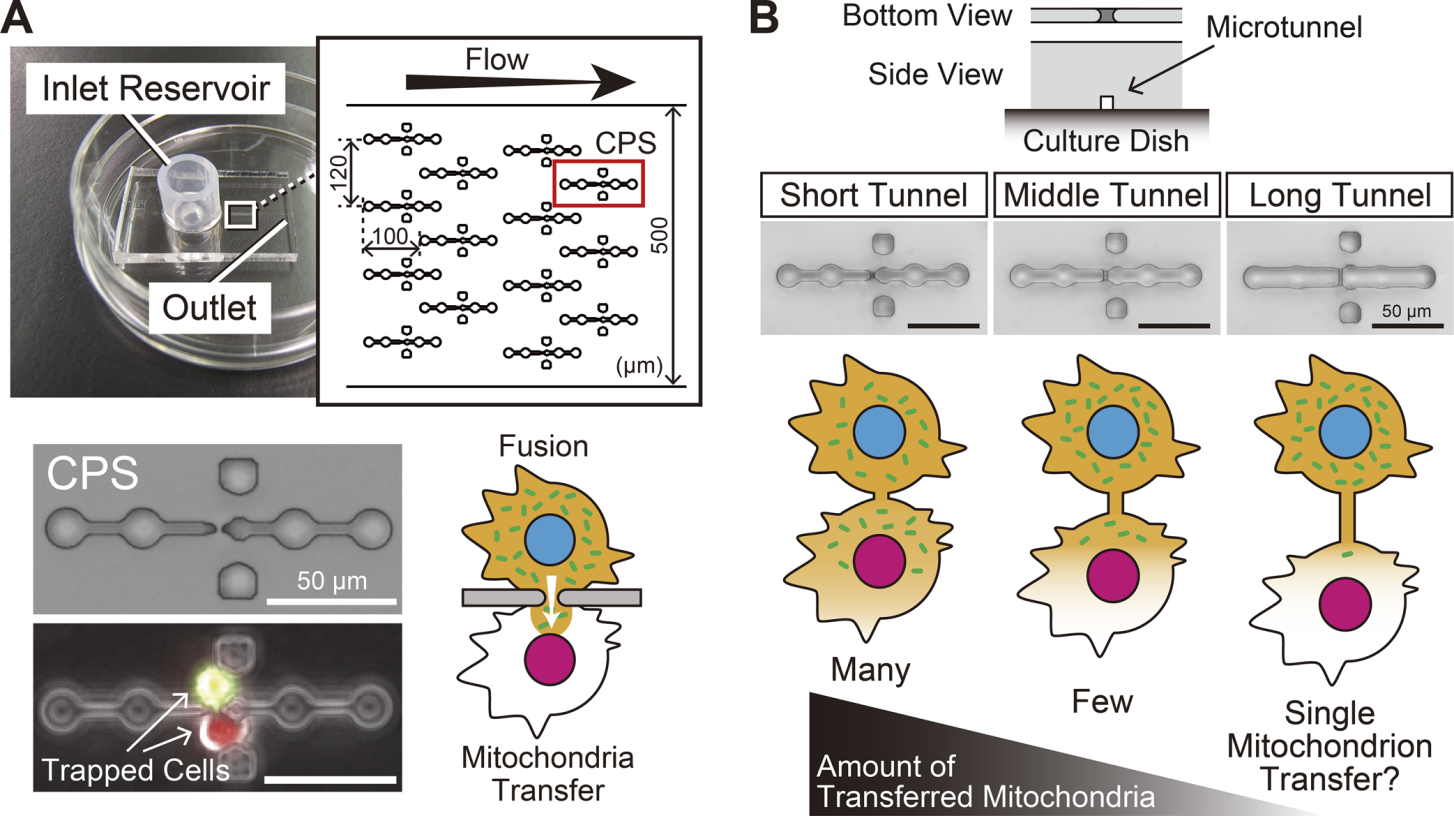
**Microfluidic device for mitochondria transfer between live single cells.** (A) The microfluidic device used for mitochondria transfer (our previous microfluidic device). In the main microchannel, a total of 105 cell pairing structures (CPSs), which can trap single cells in a pairwise manner at the position of the microaperture (microslit), are arrayed. Cell fusion through a microslit produces a strictured cytoplasmic connection which allows migration of cytoplasmic components including mitochondria into the fusion partner. In the present study, the microslit was replaced with a microtunnel (see panel B). Data are from references (Wada et al., 2014, 2015). (B) Strategy for quantitative control of mitochondria transfer. Upper panels: newly fabricated CPSs, which have a short, middle or long tunnel instead of a microslit. Lower scheme: the concept of quantitative control of mitochondria transfer. We expected that cell fusion through a microtunnel of a different length would result in formation of a strictured cytoplasmic connection with an analogous length, and that a longer cytoplasmic connection would result in fewer mitochondria being transferred including single mitochondrion transfer.

We previously developed a novel mitochondria transfer method using a microfluidic device in which paired single cells were fused through a microslit to promote a strictured cytoplasmic connection. In this situation, mitochondria gradually migrated to the fusion partner segregated from the nucleus (Fig. 1A) (Wada et al., 2014, 2015). We consequently hypothesized that elongating the length of the strictured cytoplasmic connection would result in fewer mitochondria being transferred because of difficulty in passing through the connection. In other words, modulation of the length of the strictured cytoplasmic connection would lead to quantitative control of mitochondria transfer (Fig. 1B). In the present study, we aimed to develop a method for quantitative control of mitochondria transfer between live single cells for the purpose of single mitochondrion transfer according to the strategy described above.

## RESULTS

### Promotion of different lengths of strictured cytoplasmic connection between live single cells

In order to produce a strictured cytoplasmic connection of different lengths between paired single cells, we fabricated three types of microfluidic devices based on our previous microfluidic device. In the new devices, microslits in the cell paring structures (CPSs) were replaced by microtunnels with average lengths of 4.1, 5.6 and 10.0 μm (i.e. a short, middle and long tunnel, respectively) (Fig. 1B). The width and height of the microtunnel were each approximately 2 μm. We confirmed cell capture in newly fabricated microfluidic devices using Ng3T3 and CrNr3T3 cells. After cell suspension flowing, >90% of the CPSs were successfully occupied with 1:1 cell pairs, and approximately 30–40 heterogeneous cell pairs were achieved in one microfluidic device.

**Fig. 2.**
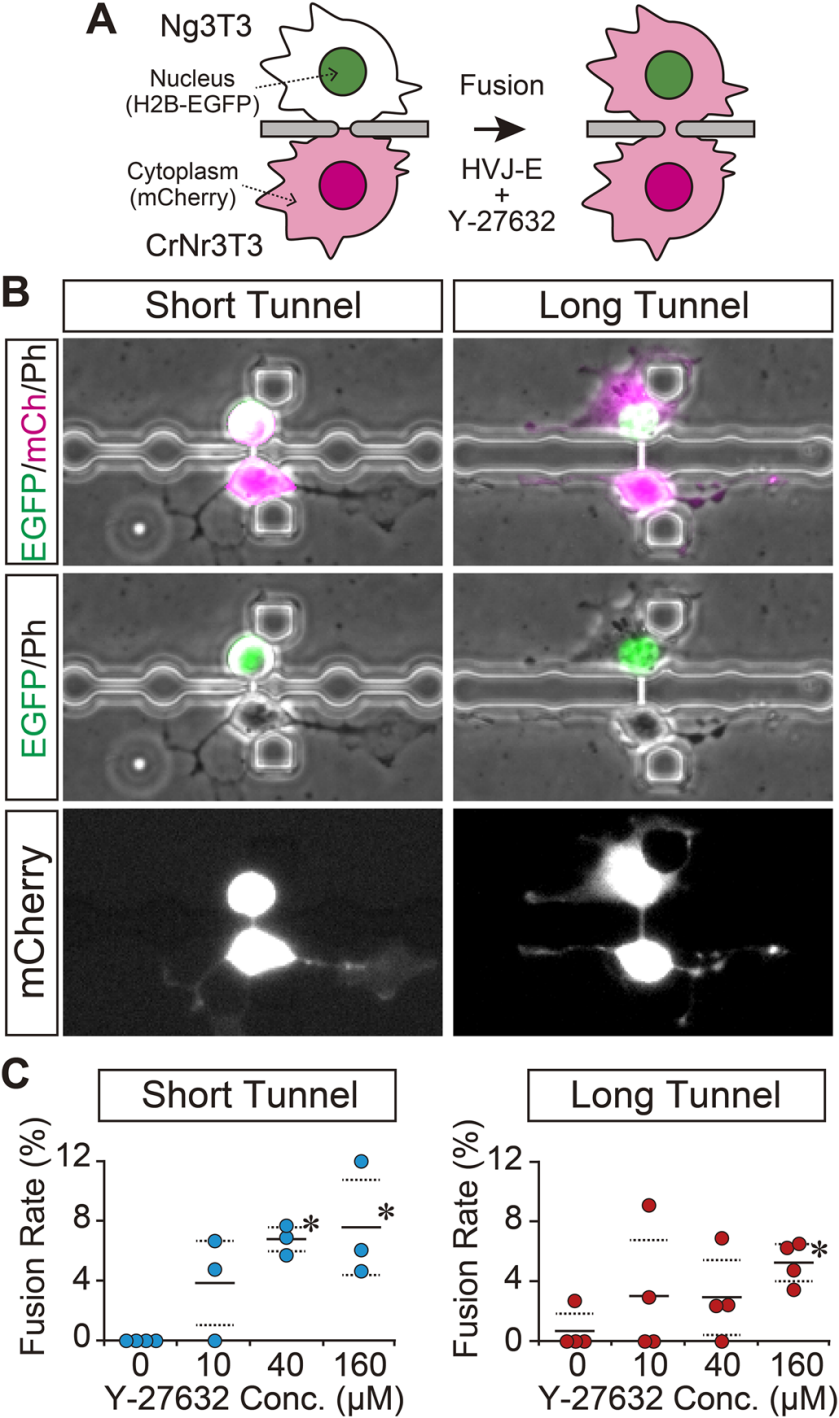
**Promotion of strictured cytoplasmic connection between live single cells.** (A) Schematic illustration of the experimental procedure. Cell fusion was induced through a microtunnel between heterogeneous pairs of Ng3T3 and CrNr3T3 cells by exposure to fusion medium with 160 μM Y-27632 for 1 h. Cell fusion was detected by transfer of mCherry from CrNr3T3 to Ng3T3 cells. (B) Achieved strictured cytoplasmic connection. Data pertaining to short and long tunnels are presented. A strictured cytoplasmic connection with a length corresponding to that of the microtunnel was successfully obtained. H2B-EGFP (EGFP: nuclei), mCherry (mCh: cytoplasm) and phase contrast (Ph) images are shown in green, magenta (or white) and gray, respectively. (C) Effect of Y-27632 supplementation on cell fusion through a microtunnel. Fusion rate in heterogeneous cell pairs of Ng3T3 and CrNr3T3 cells are presented. Each plot represents the result from one independent experiment. Horizontal solid and dashed lines represent average values and ±s.d., respectively. *Significantly different from 0 μM evaluated by Dunnett's test (*P*<0.05).

To induce cell fusion through a microtunnel, we first tested a standard Sendai virus envelope (HVJ-E) method (i.e. exposure to a culture medium containing HVJ-E without supplementation); however, that procedure rarely induced cell fusion through a microtunnel (Fig. 2C, 0 μM), perhaps due to the absence of cell–cell contact between the paired cells. Since we previously found that supplementation with Y-27632, a ROCK (Rho-associated coiled coil-forming protein kinase) inhibitor, enhanced cell fusion through a microslit (Wada et al., 2015), we then applied that protocol for the induction of cell fusion through a microtunnel. Our results confirmed that supplementation with Y-27632 led to cell fusion through a microtunnel, and thus we succeeded in achieving a strictured cytoplasmic connection between paired cells with a length corresponding to that of the microtunnel (Fig. 2B). Supplementation with Y-27632 enabled cell fusion through a microtunnel due at least partly to the promotion of neurite-like processes (Hirose et al., 1998), which are likely to result in penetration into the microtunnel to produce cell–cell contact between the paired cells. Indeed, we had shown that Y-27632 supplementation enhanced promotion of cellular protrusion from a microslit (Wada et al., 2015). Among conditions tested, a 160 μM Y-27632 supplementation was the most effective protocol for induction of cell fusion through a microtunnel. We mainly used this fusion protocol in our subsequent investigations using the NIH3T3-delived cells.

### Different lengths of cytoplasmic connection allowed quantitative control of mitochondria transfer including single mitochondrion transfer

**Fig. 3.**
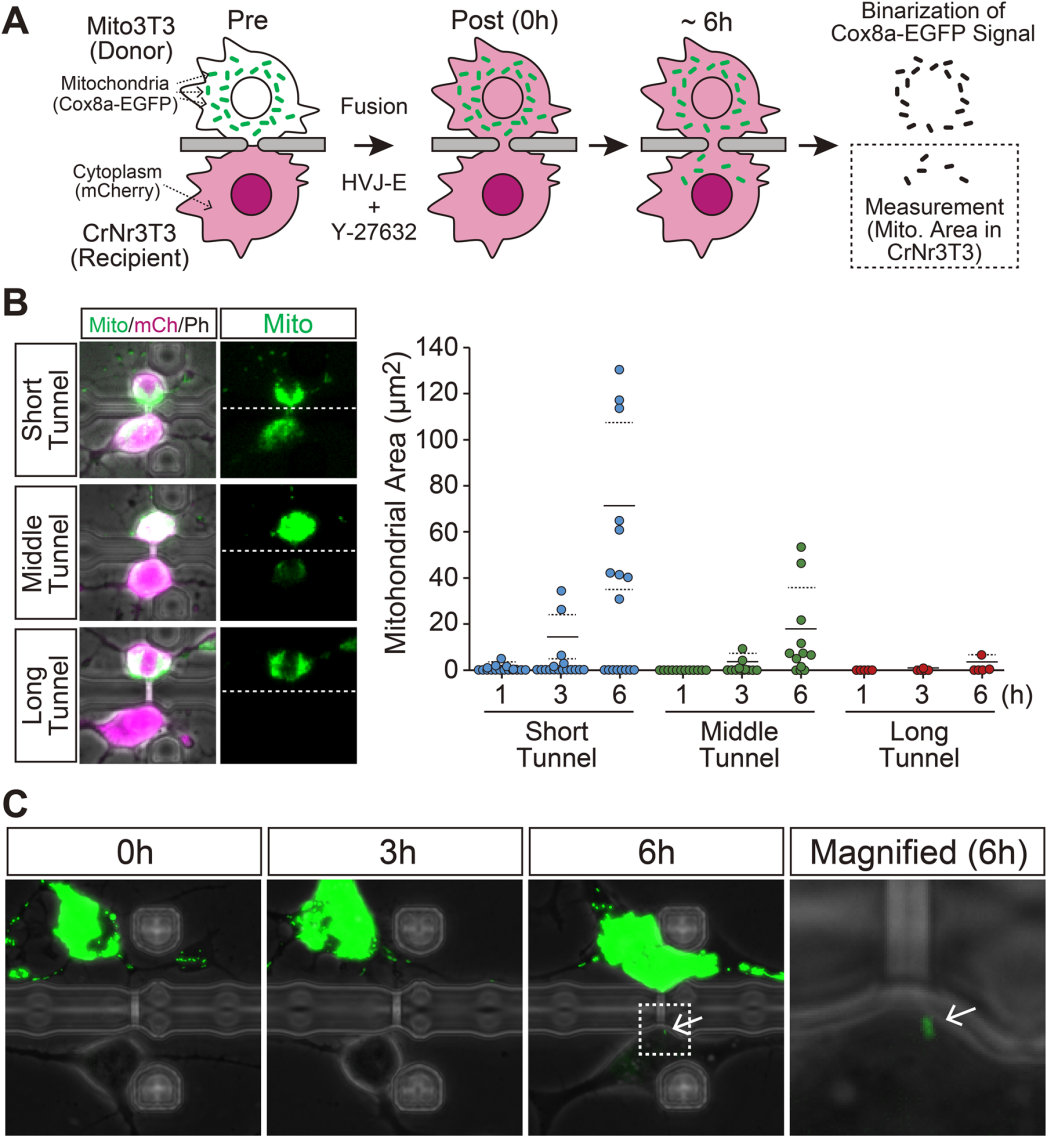
**Quantitative control of mitochondria transfer by modulating the length of the strictured cytoplasmic connection.** (A) Schematic illustration of the experimental procedure. Cell fusion was induced between Mito3T3 and CrNr3T3 cells through a microtunnel by exposure to fusion medium with 160 μM Y-27632 for 1 h. After detection of cell fusion by the transfer of mCherry from CrNr3T3 to Mito3T3 cells, the cells were incubated for 6 h without medium change. The quantity of transferred mitochondria was evaluated by measuring the Cox8a-EGFP signal area in a CrNr3T3 cell. (B) Mitochondria transfer between cell pairs fused through a microtunnel of different lengths. Left panels: examples of cell pairs fused through a long, middle or short tunnel. Dashed lines represent the location of separation wall of CPS. Cox8a-EGFP (Mito: mitochondria), mCherry (mCh: cytoplasm) and phase contrast (Ph) images are shown in green, magenta and gray, respectively. Right graph: the result of image-based quantification of transferred mitochondria. Each plot represents data from one fused pair. Same cell pairs were observed at 1, 3 and 6 h after cell fusion. Horizontal solid and dashed lines represent average values and ±s.d., respectively, those which were evaluated from the data excluding non-transferred cell pairs. (C) Timelapse observation of the cell pair fused through a long tunnel. Data is from the other set of experiments shown in panel B. Mito3T3 and CrNr3T3 cells were fused through a long tunnel by exposure to fusion medium with 160 μM Y-27632 for 3 h, then the medium was replaced with culture medium and incubated for 6 h. Arrows indicate the transferred single mitochondrion. The region of the white box is magnified in the right panel. Cox8a-EGFP (mitochondria) and phase contrast images are shown in green and gray, respectively.

We next examined whether a difference in the length of the strictured cytoplasmic connection affects the quantity of mitochondria being transferred to the fusion partner by observing fused heterogeneous pairs of Mito3T3 and CrNr3T3 cells (Fig. 3A). After 6 h from cell fusion, the Cox8a-EGFP signals were detected in CrNr3T3 cells in 53, 75 and 40% of cell pairs fused through a short, middle and long tunnel, respectively (Fig. 3B). This finding indicates that mitochondria transfer occurred at least from Mito3T3 to CrNr3T3 cells through the strictured cytoplasmic connection. Although some fused cells showed no mitochondria transfer evaluated by migration of Mito-EGFP signals, elongation of the length of the strictured cytoplasmic connection resulted in fewer mitochondria being transferred in the cell pairs showing mitochondria transfer, as we expected. In particular, a cytoplasmic connection formed through a long tunnel severely restricted mitochondria migration to the fusion partner at least during the first 6 h from induction of cell fusion. As a consequence, we detected single mitochondrion transfer in cell pairs fused through a long tunnel (Fig. 3C).

### Disconnection of fused cells

**Fig. 4.**
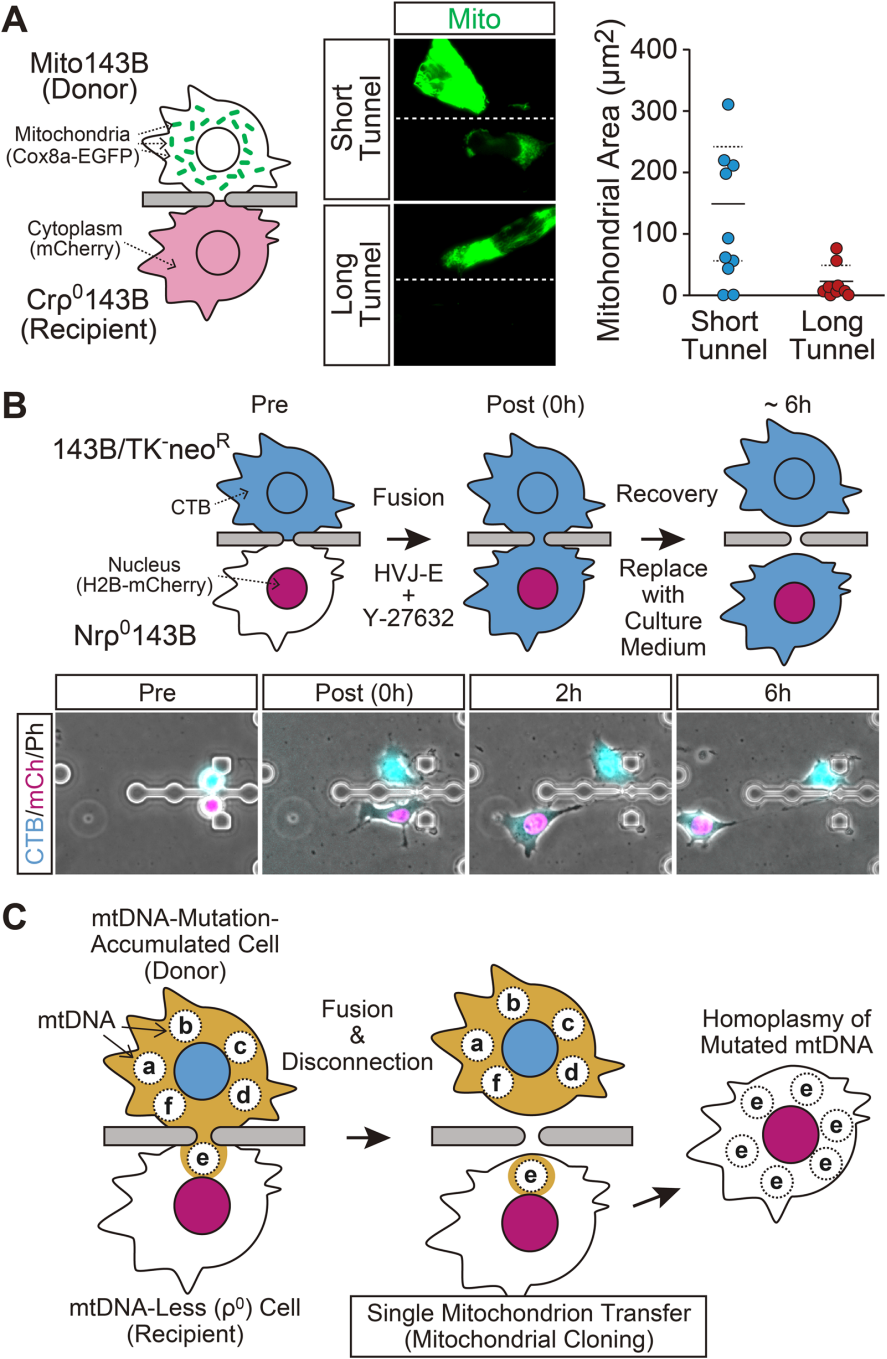
**Mitochondria transfer to a ρ^0^ cell.** (A) Quantitative control of mitochondria transfer to a ρ^0^ cell. Left: schematic illustration of the experiment. Cell fusion was induced between Mito143B and Crρ^0^143B cells through a short or long tunnel by exposure to fusion medium with 100 μM Y-27632 for 1 h. Then cells were incubated with culture medium for 6 h, and amount of mitochondria being transferred was evaluated by measuring the Cox8a-EGFP signal area in a Crρ^0^143B cell. Middle: examples of cell pairs fused through a long or short tunnel. Cox8a-EGFP (Mito: mitochondria) images are shown in green. Dashed lines represent the location of separation wall of CPS. Right graph: the result of image-based quantification of transferred mitochondria. Each plot represents data from one fused pair. Horizontal solid and dashed lines represent average values and ±s.d., respectively, those which were evaluated from the data excluding non-transferred cell pairs. (B) Disconnection of fused cells. Upper scheme: experimental procedure. 143B/TK^−^neo^R^ cells were stained with CellTracker Blue dye (CTB) prior to cell fusion. Because synergistic cytotoxicity of CTB and Y-27632 was observed, cell fusion between CTB-stained 143B/TK^−^neo^R^ and Nrρ^0^143B cells was induced by exposure to fusion medium with 40 μM (i.e. <100 μM) Y-27632 for 1 h. Then cells were incubated with culture medium for 6 h. Lower panels: an example of disconnection of fused cell pair. CTB, H2B-mCherry (mCh: nucleus) and phase contrast (Ph) images are shown in blue, magenta and gray, respectively. (C) Proposed method for introducing a homoplasmic mtDNA mutation based on single mitochondrion transfer using our microfluidic device. The scheme shows an example of obtaining homoplasmy of the mutated mtDNA, genotype ‘e’. In this scheme, mitochondria are omitted.

In order to realize mitochondrial cloning by our microfluidic device, it is essential to promote a cytoplasmic connection with a ρ^0^ cell. Furthermore, we need to develop a way to disconnect the fused cells. We found that a Y-27632 supplemented protocol successfully induced cell fusion between 143B/TK^−^neo^R^ and Nrρ^0^143B cells through a microtunnel, and mitochondria transfer to Nrρ^0^143B was observed in 80 and 89% of the fused cell pairs in the short and long tunnel, respectively. Similar to NIH3T3-derived cells, the promoted different lengths of cytoplasmic connection led to quantitative control of mitochondria transfer to a ρ^0^ cell; fewer Cox8a-EGFP signals were found in ρ^0^ cells in the cell pairs fused through a long tunnel (Fig. 4A). After cell fusion induction, the fusion medium was replaced with a normal culture medium as recovery culture. Interestingly, the fused cells were spontaneously disconnected from each other while in the recovery culture, and for some of them, the cytoplasmic disconnection occurred within 6 h from the onset of the recovery culture (Fig. 4B). Since we showed that single mitochondrion transfer occurred during that period in the NIH3T3-delived cells (Fig. 3C), this data suggest that our cell fusion system using a microfluidic device is likely to realize single mitochondrion transfer to a ρ^0^ cell.

## DISCUSSION

In this study, we developed microfluidic devices having different lengths of a microtunnel, and succeeded in producing different lengths of strictured cytoplasmic connection between live single cells by cell fusion through the microtunnel. We found that elongation of the length of the strictured cytoplasmic connection restricted mitochondria transfer, and demonstrated that a cytoplasmic connection formed through a long tunnel led to single mitochondrion transfer. Moreover, we succeeded in achieving transient cytoplasmic connection with a ρ^0^ cell through the microtunnel. Overall data suggest that our cell fusion system provides a promising approach to perform single mitochondrion transfer to a ρ^0^ cell. Based on this, we here propose a novel mtDNA manipulation method using a microfluidic device which can achieve homoplasmic mtDNA mutation(s) (Fig. 4C). At first, random mtDNA mutations are introduced to donor cells by chemical treatment (Marcelino and Thilly, 1999) or biological method (Spelbrink et al., 2000; Trifunovic et al., 2004). A single mitochondrion is then transferred from the donor to a ρ^0^ cell. This process leads to cloning of mutated mtDNA in the ρ^0^ cell if the transferred single mitochondrion contains the same population of mutated mtDNA (i.e. a nucleoid composed of the same mtDNA genotype), and homoplasmy of the mutated mtDNA is established after recovery of the mtDNA.

For the mtDNA manipulation method proposed above, single mitochondrion transfer to a ρ^0^ cell is the most important key process. Mitochondria transfer to live cells can be performed through some methods other than our cell-fusion-based method. In particular, microinjection of isolated mitochondria can be used to perform single mitochondrion transfer (King and Attardi, 1988); however, microinjection not only requires special skills for precise manipulation, but also risks causing severe damage to the cells, resulting in a low throughput. Previous studies have reported that simple co-culture can induce mitochondria transport between neighboring cells by an intrinsic mechanism (Spees et al., 2006; Cho et al., 2012; Lou et al., 2012). This cellular behavior leads to noninvasive mitochondria transfer; however, it occurs only between specific cells and the quantity of mitochondria transportation is uncontrollable. In contrast to the aforementioned methods, our microfluidic device performs quantitative control of mitochondria transfer including single mitochondrion transfer using a very simple procedure, and can be applied to a wide range of adhesive cells. Since there are no reliable studies reporting mtDNA heterogeneity within single nucleoid and we have no data about successful repopulation rate of imported single mitochondrion, it is difficult to estimate precise probability of occurrence of homoplasmy in our system; however, even if the probability of occurrence of homoplasmy is low, our device would overcome this problem because throughput of the microfluidic device can be easily raised by increasing the CPS number. Although we have not yet succeeded in achieving homoplasmy of mutated mtDNA in ρ^0^ cells, our cell fusion method using a microfluidic device provides a promising approach to achieve homoplasmy of mutated mtDNA, and thus will be a unique and innovative platform for mitochondria genetics.

## MATERIALS AND METHODS

### Cells and cell culture

The NIH3T3 cell-derived cell lines Ng3T3 (nucleus is labeled with H2B-EGFP), CrNr3T3 (cytoplasm and nucleus are labeled with mCherry and H2B-mCherry, respectively) and Mito3T3 (mitochondria are labeled with Cox8a-EGFP) were previously established (Wada et al., 2014, 2015). The 143B/TK^−^neo^R^ cells (Pereira-Smith and Smith, 1988) were provided by the RIKEN BRC through the National Bio-Resource Project of MEXT, Japan. The 143B/TK^−^neo^R^ cell-derived cell line Mito143B (mitochondria are labeled with Cox8a-EGFP) was established by stable transfection of Cox8a-EGFP expression vector derived from pcDNA-Mito-EGFP (Abe et al., 2011). The mtDNA-less (ρ^0^) cell line Crρ^0^143B (cytoplasm is labeled with mCherry) and Nrρ^0^143B (nucleus is labeled with H2B-mCherry) were established from 143B/TK^−^neo^R^ cells by elimination of mtDNA followed by stable transfection of mCherry and H2B-mCherry expression vector derived from pcDNA3-H2B-mCherry (Abe et al., 2011), respectively. The mtDNA was eliminated from 143B/TK^−^neo^R^ cells by ethidium bromide (EtBr) exposure (King and Attardi, 1989). The 143B/TK^−^neo^R^ cells were cultured for 56 days with a culture medium supplemented with 50 ng/ml EtBr, 50 μg/ml uridine and 100 μg/ml pyruvate. The cells were then further cultured for 33 days without EtBr supplementation, and the absence of mtDNA in cells was confirmed by a PCR method.

The cells were cultured with Dulbecco's modified Eagle's medium containing 4500 mg/l glucose (Wako Pure Chemical Industry Ltd., Osaka, Japan) supplemented with 10% fetal bovine serum. For the Nrρ^0^143B and Crρ^0^143B cells culture, 50 μg/ml uridine and 100 μg/ml pyruvate were further added to the culture medium.

### Microfluidic devices

The microfluidic devices were fabricated using a photolithography-based method as described elsewhere with some modifications (Wada et al., 2014). Briefly, the master molds for a poly(dimethylsiloxane) (PDMS) chip, which have 105 CPSs with a microtunnel, were fabricated on Si wafers using SU-8 (MicroChem Corp., MA) by well-aligned double exposure of micro-patterned UV light. The first and second exposure was used for optical modeling of the microtunnel structure and main channel region, respectively. After curing of PDMS on the master mold at 65°C >1 h, the PDMS chip was harvested and trimmed. A silicon tube with an inner diameter of 5 mm was then connected to make an inlet reservoir. The resulting PDMS chip was reversibly bonded to the bottom of a 35-mm culture dish to make the microfluidic devices.

### Cell fusion and live cell imaging

Cell fusion through a microtunnel was induced as previously described (Wada et al., 2015). Briefly, after making cell pairs by hydrodynamic trapping in the microdevice, a culture medium containing a 1/10 volume of HVJ-E suspension (Ishihara Sangyo Kaisha Ltd., Osaka, Japan) supplemented with 0-160 μM Y-27632 (fusion medium) was injected from the inlet reservoir, and the apparatus was then incubated in a CO_2_ chamber for 1-3 h, and the fluorescent signal was directly observed by reverse fluorescent microscopy to detect cell fusion. Cell fusion was detected by migration of mCherry from CrNr3T3/Crρ^0^143B cells to Ng3T3/Mito3T3/Mito143B cells. In the experiment using 143B/TK^−^neo^R^ and Nrρ^0^143B cells, 143B/TK^−^neo^R^ cells were stained with 40 μM CellTracker Blue dye (CTB) (Thermo Fisher Scientific Inc., MA) for 15 min prior to the cell fusion, and the CTB signal was observed to detect cell fusion.

### Data analysis

Image-based quantification was performed using ImageJ ver. 1.45s software (National Institute of Mental Health, MD). Statistical data were analyzed by one-way ANOVA followed by Dunnett's test using KaleidaGraph ver. 4.5 (Synergy Software, Reading, PA). A value of *P*<0.05 was regarded as significant.
